# Worsened restrictive lung physiology due to acute colonic pseudo-obstruction

**DOI:** 10.1093/omcr/omac106

**Published:** 2022-10-22

**Authors:** Kyle Admire, Nikhil Shah, Priscilla Yee, Maria Cirino-Marcano

**Affiliations:** Department of Internal Medicine, Virginia Tech – Carilion, Roanoke, VA, USA; Department of Internal Medicine, Virginia Tech – Carilion, Roanoke, VA, USA; Department of Pulmonary and Critical Care Medicine, Virginia Tech – Carilion, Roanoke, VA, USA; Department of Pulmonary and Critical Care Medicine, Virginia Tech – Carilion, Roanoke, VA, USA

**Figure 1:**
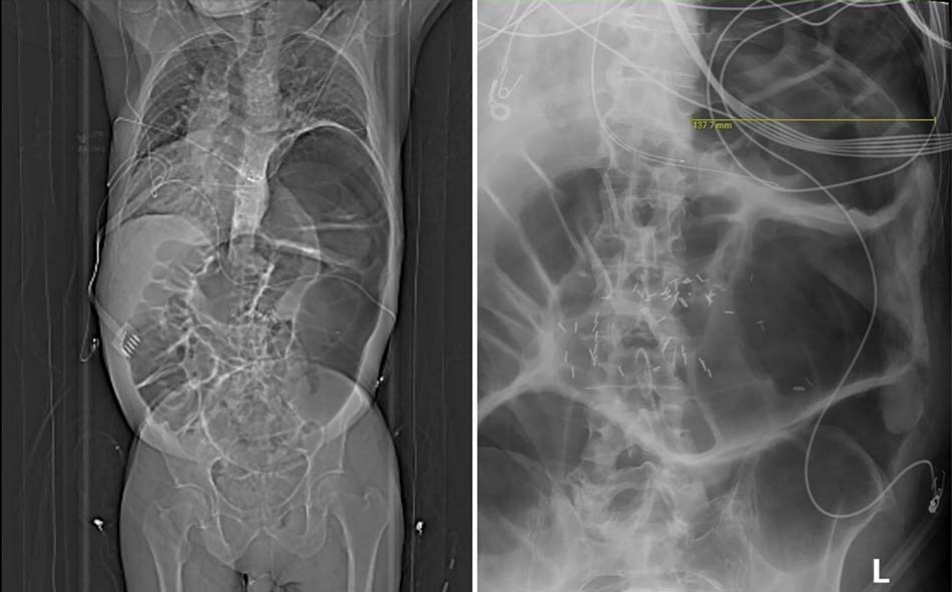
Scout CT image showing severe colonic dilation causing cranial displacement of the left hemidiaphragm and unilateral compression of the left thorax; abdominal radiograph after the second administration of neostigmine showing persistent colonic dilation measuring 13.8 cm.

A 76-year-old man with left-hemidiaphragm paralysis after left diaphragm eventration during childhood surgical repair of a patent ductus arteriosus with resultant restrictive lung disease and recent bunionectomy with calcaneal autograft presented to the emergency department with dyspnea and hypoxemia without leukocytosis. Initial evaluation showed hypoxemia without leukocytosis. CTA-chest did not reveal a pulmonary embolus but showed left-hemidiaphragm elevation and colonic distention (Fig. 1). Subsequent abdominal imaging redemonstrated this finding. After admission, he developed new confusion and respiratory distress. Arterial blood gas on nasal cannula revealed worsened hypoxemia and new hypercapnia, requiring intubation and mechanical ventilation. CT with rectal contrast was consistent with acute colonic pseudo-obstruction (Ogilvie’s Syndrome). He underwent treatment with aggressive electrolyte repletion, multiple tap-water enemas, and two administrations of neostigmine which did not resolve the pseudo-obstruction. Gastroenterology performed colonoscopic decompression with decompression tube placement, resulting in resolution of colonic distention.

Unilateral hemidiaphragm paralysis is a well-documented complication of cardiothoracic surgeries with up to 20% of cases reporting at least temporary palsy following the procedure [1]. Those with hemidiaphragm paralysis may exhibit restrictive lung physiology on spirometry, as did our patient. Ogilvie’s syndrome is a clinical syndrome characterized by the signs and symptoms of mechanical obstruction without such a cause. There is no known mechanism for this condition, but it is presumed to be due to an unknown autonomic disturbance in parasympathetic function [2]. In general, the diaphragm should limit the amount of possible abdominal distension. In our patient, the over-distension of the abdomen caused a cranial shift of the affected portions of the diaphragm. Pelosi and colleagues [3] demonstrated that cranial displacement of the diaphragm reduces chest wall compliance and overall lung volume. Our case illustrates a unique circumstance in which a patient was predisposed to acute respiratory failure due to diaphragm paralysis and colonic distention.
